# Checklist of the families Anisopodidae, Bibionidae, Canthyloscelididae, Mycetobiidae, Pachyneuridae and Scatopsidae (Diptera) of Finland

**DOI:** 10.3897/zookeys.441.7358

**Published:** 2014-09-19

**Authors:** Antti Haarto

**Affiliations:** 1 Zoological Museum, Section of Biodiversity and Environmental Science, Department of Biology, FI-20014 University of Turku, Finland

**Keywords:** Checklist, Finland, Diptera, biodiversity, faunistics

## Abstract

A checklist of the families Anisopodidae, Bibionidae, Canthyloscelididae, Mycetobiidae, Pachyneuridae and Scatopsidae (Diptera) recorded from Finland. The Finnish species of these families were last listed in 1980. After that two species of Anisopodidae, four species of Bibionidae and six species of Scatopsidae have been added to the Finnish fauna and two species of Scatopsidae have been removed from the Finnish fauna.

## Introduction

Six families (Anisopodidae, Bibionidae, Canthyloscelididae, Mycetobiidae, Pachyneuridae and Scatopsidae) are included in this paper. All six families have been rather poorly collected and studied in Finland. Lundström (1910) published a study of the family Bibionidae. During the last one hundred years, foreign specialists have contributed the majority of the new records and taxonomic treatises. Some Anisopodidae were treated by Krivosheina and Menzel (1998, 2002) and Bibionidae by Greve and Haenni (1994) and Skartveit (1999). Finnish scatopsid records were included in the synopsis of Palaearctic Scatopsidae by Cook (1969, 1972, 1974). The genera *Anapausis* Enderlein (Chandler 1999, Haenni 1984), *Colobostema* Enderlein (Haenni 2013, Krivosheina 2001) and *Aspistes* Meigen (Krivosheina 2000) have been examined in more detail. Recently Anisopodidae (Haarto 2011) and Bibionidae (Haarto 2012) have attracted some attention in the country. The only Finnish species of Pachyneuridae, *Pachyneura fasciata* Zetterstedt, 1838, was studied by Salmela and Ilmonen (2004) and removed from the Finnish Red List. The Finnish species of all six families were last listed by Hackman (1980). The Finnish faunae of anisopodids and bibionids have been recently reviewed by Haarto (2011, 2012). The nomenclature and the number of European species are presented according to the current listing of Fauna Europaea (Beuk and Pape 2013).

**Table 1.** Number of species by family.

Family	Number of species in			Level of knowledge
	World (Pape et al. 2011)	Europe	Finland	
Anisopodidae	77	10	7	average
Mycetobiidae	35	4	1	average
Bibionidae	760	50	17	average
Scatopsidae	390	102	30	poor
Canthyloscelididae	16	3	3	average
Pachyneuridae	5	1	1	average

## Checklist

Nematocera Duméril, 1805

infraorder Bibionomorpha Hennig, 1954

superfamily Anisopodoidea Knab, 1912

**ANISOPODIDAE** Knab, 1912

***SYLVICOLA*** Harris, 1780

= ***Phryne*** Meigen, 1800 suppr.

= ***Rhyphus*** Latreille, 1804

*Sylvicola cinctus* (Fabricius, 1787)

*Sylvicola fenestralis* (Scopoli, 1763)

*Sylvicola fuscatoides* Michelsen, 1999

= *fuscatus* sensu Andersson 1967, nec Fabricius

*Sylvicola fuscatus* (Fabricius, 1775)

*Sylvicola punctatus* (Fabricius, 1787)

*Sylvicola stackelbergi* Krivosheina & Menzel, 1998

*Sylvicola zetterstedti* (Edwards, 1923)

**MYCETOBIIDAE** Winnertz, 1863

***MYCETOBIA*** Meigen, 1818

*Mycetobia pallipes* Meigen, 1818

superfamily Bibionoidea Fleming, 1821

**BIBIONIDAE** Fleming, 1821

BIBIONINAE Fleming, 1821

***BIBIO*** Geoffroy, 1762

*Bibio brunnipes* (Fabricius, 1794)

= *fulvipes* (Zetterstedt, 1838)

*Bibio clavipes* Meigen, 1818

*Bibio ferruginatus* (Linnaeus, 1767)

*Bibio fulvicollis* Gimmerthal, 1842

= *festinans* (Zetterstedt, 1850)

*Bibio johannis* (Linnaeus, 1767)

*Bibio lautaretensis* Villeneuve, 1925

= *crassipes* Duda, 1930

*Bibio longipes* Loew, 1864

= *lepidus* Loew, 1871

*Bibio marci* (Linnaeus, 1758)

*Bibio nigriventris* Haliday, 1833

*Bibio pomonae* (Fabricius, 1775)

*Bibio rufipes* (Zetterstedt, 1838)

*Bibio siebkei* Mik, 1887

= *femoralis* (Siebke, 1863) preocc.

*Bibio varipes* Meigen, 1830

***DILOPHUS*** Meigen, 1803

*Dilophus borealis* Skartveit, 1993

*Dilophus febrilis* (Linnaeus, 1758)

*Dilophus femoratus* Meigen, 1804

PLECIINAE Fleming, 1821

***PENTHETRIA*** Meigen, 1803

*Penthetria funebris* Meigen, 1804

= *holosericea* Meigen, 1818

superfamily Scatopsoidea Newman, 1834

**SCATOPSIDAE** Newman, 1834

ASPISTINAE Rondani, 1840

***ARTHRIA*** Kirby in Richardson, 1837

*Arthria analis* (Kirby in Richardson, 1837)

***ASPISTES*** Meigen, 1818

*Aspistes berolinensis* Meigen, 1818

*Aspistes freyi* Cook, 1965

*Aspistes helleni* Krivosheina, 2000

ECTAETIINAE Enderlein, 1936

***ECTAETIA*** Enderlein, 1912

*Ectaetia clavipes* (Loew, 1846)

*Ectaetia platyscelis* (Loew, 1869)

PSECTROSCIARINAE Cook, 1963

***ANAPAUSIS*** Enderlein, 1912

*Anapausis floricola* Chandler, 1999

= *soluta* misid.

*Anapausis rectinervis* Duda, 1928

SCATOPSINAE Newman, 1834

tribe Colobostematini Amorim, 1994

***COLOBOSTEMA*** Enderlein, 1926

*Colobostema infumatum* (Haliday, 1833)

*Colobostema nigripenne* (Meigen, 1830)

*Colobostema triste* (Zetterstedt, 1850)

***EFCOOKELLA*** Haenni, 1998

*Efcookella albitarsis* (Zetterstedt, 1850)

***FERNEIELLA*** Cook in Freeman, 1985

*Ferneiella incompleta* (Verrall, 1886)

***HOLOPLAGIA*** Enderlein, 1912

*Holoplagia bullata* (Edwards, 1925)

*Holoplagia transversalis* (Loew, 1846)

tribe Rhegmoclematini Cook, 1955

***RHEGMOCLEMINA*** Enderlein, 1936

*Rhegmoclemina vaginata* (Lundström, 1910)

***THRIPOMORPHA*** Enderlein, 1905

= ***Rhegmoclema*** Enderlein, 1912

*Thripomorpha freyi* (Cook, 1969)

*Thripomorpha halteratum* (Meigen, 1838)

*Thripomorpha paludicola* Enderlein, 1905

= *edwardsi* (Collin, 1954)

*Thripomorpha verralli* (Edwards, 1934)

tribe Scatopsini Newman, 1834

***APILOSCATOPSE*** Cook, 1974

*Apiloscatopse flavicollis* (Meigen, 1818)

*Apiloscatopse flavocincta* (Duda, 1928)

***REICHERTELLA*** Enderlein, 1912

*Reichertella geniculata* (Zetterstedt, 1850)

***SCATOPSE*** Geoffroy, 1762

*Scatopse lapponica* Duda, 1928

*Scatopse notata* (Linnaeus, 1758)

tribe Swammerdamellini Cook, 1972

***COBOLDIA*** Melander, 1916

*Coboldia fuscipes* (Meigen, 1830)

***RHEXOZA*** Enderlein, 1936

*Rhexoza subnitens* (Verrall, 1886)

***SWAMMERDAMELLA*** Enderlein, 1912

*Swammerdamella adercotris* Cook, 1972

*Swammerdamella brevicornis* (Meigen, 1830)

*Swammerdamella genypodis* Cook, 1972

**CANTHYLOSCELIDIDAE** Enderlein, 1912

CANTHYLOSCELIDINAE Enderlein, 1912

***HYPEROSCELIS*** Hardy & Nagatomi, 1960

*Hyperoscelis eximia* (Boheman, 1858)

*Hyperoscelis veternosa* Mamaev & Krivosheina, 1969

SYNNEURINAE Enderlein, 1936

***SYNNEURON*** Lundström, 1910

*Synneuron annulipes* Lundström, 1910

superfamily Pachyneuroidea Schiner, 1864

**PACHYNEURIDAE** Schiner, 1864

***PACHYNEURA*** Zetterstedt, 1838

*Pachyneura fasciata* Zetterstedt, 1838

## Excluded species

*Anapausis soluta* (Loew, 1846): Specimens under the name *Anapausis soluta* in the earlier checklist (Hackman 1980) represent the later described species *Anapausis floricola* Chandler, 1999.

*Swammerdamella acuta* Cook, 1956: Has not been found within present borders of Finland.

## Notes

***Aspistes helleni* Krivosheina, 2000** in this checklist based on other material than the one mentioned by Krivosheina (2000).
